# Iota-carrageenan and xylitol inhibit SARS-CoV-2 in Vero cell culture

**DOI:** 10.1371/journal.pone.0259943

**Published:** 2021-11-19

**Authors:** Shruti Bansal, Colleen B. Jonsson, Shannon L. Taylor, Juan Manuel Figueroa, Andrea Vanesa Dugour, Carlos Palacios, Julio César Vega

**Affiliations:** 1 Regional Biocontainment Laboratory, University of Tennessee Health Science Center, Memphis, Tennessee, United States of America; 2 Department of Microbiology, Immunology and Biochemistry, The University of Tennessee Health Science Center, Memphis, Tennessee, United States of America; 3 LogixBio, Holly Springs, North Carolina, United States of America; 4 Respiratory Research Group, Instituto de Ciencia y Tecnología Dr. César Milstein—(Consejo Nacional de Investigaciones Científicas y Técnicas CONICET- Fundación Pablo Cassará), Ciudad de Buenos Aires, Argentina; 5 Department of Research and Development, Amcyte Pharma Inc., Cambridge, Massachusetts, United States of America; University of Washington, UNITED STATES

## Abstract

Last year observed a global pandemic caused by SARS-CoV-2 (severe acute respiratory syndrome-coronavirus 2) infection affecting millions of individuals worldwide. There is an urgent unmet need to provide an easily producible and affordable medicine to prevent transmission and provide early treatment for this disease. Since the nasal cavity and the rhinopharynx are the sites of initial replication of SARS-CoV-2, a nasal spray may be an effective option to target SARS-CoV-2 infection. In this study, we tested the antiviral action of three candidate nasal spray formulations against SARS-CoV-2 *in vitro*. We determined that iota-carrageenan in concentrations as low as 6 μg/mL inhibits SARS-CoV-2 *in vitro*. The concentrations of iota-carrageenan with activity against SARS-CoV-2 *in vitro* may be easily achieved through the application of nasal sprays as commonly used in several countries. Recently a double-blind, placebo-controlled study showed that iota-carrageenan in isotonic sodium chloride reduces ca. five times the risk of infection by SARS-CoV-2 in health care personnel. Further, xylitol at a concentration of 50 mg/mL (ca. 329 mM) was found to exert some antiviral action, though this preliminary finding needs further confirmation.

## Introduction

SARS-CoV-2 is the single-stranded positive-sense RNA virus responsible for COVID-19, causing one of the most significant pandemics of our time, with more than 244,980,203 confirmed cases and more than 4,971,409 deaths worldwide as of October 27^th^, 2021 [1]. In most cases, COVID-19 manifests with flu-like symptoms and results in manageable symptoms that resolve without intervention. However, 15% of patients develop severe pneumonia that requires hospitalization and oxygen support. This includes 5% requiring admission to an intensive care unit (ICU), and among these cases, half result in death [2]. Older adults and those with pre-existing conditions are most susceptible to adverse outcomes. Children are also affected but display milder symptoms than adults, nonetheless, they remain active transmitters of COVID-19 [3].

Currently, there are no adequate therapeutic or preventive medicines available for COVID-19, except for vaccines. However, by the time of this publication, only 3.1% of people in low-income countries have received at least one dose [4]. Therefore, effective approaches are urgently needed to reduce the spread of the virus and its death toll. Recent data have shown that a high viral load and a long virus-shedding period were associated with severe COVID-19 [5, 6]. Furthermore, in the early stage of pathogenesis, the virus is localized mainly in the nasal cavity and the nasopharynx [7, 8]. Therefore, the use of antiviral nasal sprays may help reduce nasal and nasopharyngeal viral load, thereby slowing down disease progression and transmission.

Iota-carrageenan formulated into a nasal spray has proven to be safe and effective against coronavirus virus causing common cold [9–11]. Moreover, iota-carrageenan-containing nasal sprays are currently available in several countries in the world. Carrageenans are linear sulfated polysaccharides that are often extracted from red seaweeds, and commercially available in the form of kappa (κ), iota (ι), or lambda (λ). They have been used for years as thickening agents and stabilizers for food and in the cosmetic and pharmaceutical industry as suspension and emulsion stabilizers. Their antiviral capacity was discovered decades ago and has been experimentally confirmed on herpes virus type 1 and 2, human papilloma virus, H1N1 influenza virus, dengue virus, rhinovirus, hepatitis A virus, enteroviruses, and coronaviruses. Iota-carrageenan inhibits several viruses based on its interaction with the surface of viral particles, thus preventing viral entry and viral budding. [12–17]. *In vitro* studies examining HeLa cells and primary respiratory epithelial cells have shown inhibition of rhinovirus and influenza. Further, in one study, iota-carrageenan spray reduced mortality by at least 50% in mice infected with lethal doses of the H1N1 influenza virus [18]. In all cases, the antiviral action of iota-carrageenan is more effective, when administrated prophylactically or in the early stages of disease and has shown synergy with other antiviral agents. Studies performed on adults and children with a common cold demonstrate the effectiveness of an iota-carrageenan nasal spray to alleviate clinical symptoms and shorten their duration, as well as to decrease the viral load of nasopharyngeal specimens and relapses during the follow-up period [9–11, 19–21].

In addition, xylitol is a polyol (formula (CHOH)_3_(CH_2_OH)_2_) that has been used as a sugar substitute in Finland since the 1960s. Obtained from xylan, which is first extracted from hardwood, xylitol has demonstrated multiple health benefits [22]. It has been extensively used in oral health care to prevent cavities because of its antibacterial capacity. It is already being used in otorhinolaryngology as a nasal spray and irrigation for the treatment of rhinosinusitis and the prevention of otitis media [23, 24]. Both *in vitro* and animal model studies have demonstrated the antiviral properties of xylitol against the human respiratory syncytial virus [25].

Both iota-carrageenan and xylitol are safe for humans, being used in much larger amounts as food additives than what may be used for nasal delivery. The safety of iota-carrageenan has already been tested intranasally in New Zealand rabbits in daily doses up to 448 μg/kg/day for up to 28 days and by inhalation in F344 rats for seven days in doses up to 1.2 mg/kg/day [26]. These studies showed neither local nor systemic toxicity. No immunotoxicity or immunogenicity was observed either. On the other hand, a 50 mg/mL xylitol aqueous solution was well tolerated when administered as a nasal irrigation to chronic rhinosinusitis patients [27] and when administered by inhalation to naïve and atopic mice, as well as to healthy human volunteers [28]. Both are included in nasal formulations currently available for use in children and adults.

Based on the above knowledge, an experiment was designed and carried out in a Biosafety Level 3 (BSL3) laboratory to investigate the SARS-CoV-2 inhibition capacity of three different candidate preservative-free formulations. It is postulated that the antiviral pathways of iota-carrageenan are due to the electrostatic attraction between its negatively charged molecules and positively charged viral particles [29]. Therefore, by increasing the ionic strength of the medium, the *in vitro* antiviral action of iota-carrageenan should decrease, as has been previously observed [30]. For this reason, we tested formulations with two different concentrations of sodium chloride (9 and 5 mg/mL) and one that contains xylitol (50 mg/mL) with almost no addition of electrolytes.

## Materials and methods

### Working conditions and installations

All work involving SARS-CoV-2 was performed at the University of Tennessee Health Science Center (UTHSC) Regional Containment Facility using BSL3 practices under protocols reviewed and approved under Institutional Biosafety Committee (IBC) 17/531.

### Cells and virus

African green monkey kidney Vero E6 cells were purchased from the American Type Culture Collection (ATCC^®^ CRL-1586^TM^). Vero E6 cells were grown in complete minimal essential media (c-MEM) (Corning, NY, USA), which included 5% fetal bovine serum (FBS) (Gibco, Waltham, MA, USA), 5 mM penicillin/streptomycin (Gibco, Waltham, MA, USA), and L-glutamine (Gibco, Waltham, MA, USA). Cells were incubated at 37°C with 5% CO_2_.

SARS-CoV-2 isolate USA-WA1/2020, was isolated from an oropharyngeal swab from a patient with a respiratory illness who had recently returned from travel to the affected region of China and developed clinical disease (COVID-19) in January 2020 in Washington, USA, it was obtained from BEI Resources, established by the National Institute of Allergy and Infectious Diseases (NIAID) to provide reagents (catalogue number NR-52281, Manassas, VA, USA). Viral master seed stock was prepared by infecting T-175 flasks of Vero E6 cells using a multiplicity of infection (MOI) of 0.1. Each flask was harvested on day two post-infection. The supernatant of each flask was centrifuged twice at 220 *x g* for 15 minutes to remove cellular debris in a benchtop centrifuge using buckets with biocontainment. Titer of virus stock was determined by plaque assay on Vero E6 cells and expressed as plaque-forming units per ml (pfu/ml) [31].

### Preparation of sample formulations

All the samples were prepared at Laboratorio Pablo Cassará S.R.L. (Argentina) under aseptic conditions and provided by Amcyte Pharma Inc. (US) to the University of Tennessee Health Science Center. The composition of the samples is depicted in Tables 1 and 2.

**Table 1:** Composition of samples containing iota-carrageenan.

Component	sample 1	sample 2	sample 3
Iota-carrageenan	1.7 mg/mL	1.2 mg/mL	1.2 mg/mL
Sodium Chloride	9 mg/mL	5 mg/mL	___
Xylitol	____	____	50 mg/mL^a^
pH adjusted to	6.00–7.00	6.00–7.00	6.00–7.00

^a^Equivalent to ca. 329 mM.

**Table 2:** Composition of samples used as diluents (samples without iota-carrageenan).

Component	sample P1	sample P2	sample P3
Sodium Chloride	9 mg/mL	5 mg/mL	____
Xylitol	____	____	50 mg/mL^a^
pH adjusted to	6.00–7.00	6.00–7.00	6.00–7.00

^a^Equivalent to ca. 329 mM.

Samples P1, P2, and P3 are referred to as Diluents P1, P2, and P3, respectively, throughout the rest of the text.

#### Preparation of formulations from Samples 1 and P1

A 1,200 μg/mL iota-carrageenan stock solution was obtained by taking 7 mL of Sample 1 and bringing it to 10 mL with Sample P1. This stock solution was used to obtain the highest final concentration of iota-carrageenan in the corresponding wells (600 μg/mL). This was achieved by applying 0.5 mL with the same volume of culture media.

Stock solutions with 120, 12, and 1.2 μg/mL iota-carrageenan concentrations were obtained from the 1,200 μg/mL stock solution by 1:10 serial dilutions using Sample P1 as diluent. The corresponding final concentrations of iota-carrageenan in the wells (60, 6, and 0.6 μg/mL) were obtained by applying 0.5 mL of each of these dilutions with the same volume of culture media.

All concentrations and control were tested in triplicate.

#### Preparation of formulations from Samples 2 and P2

The highest iota-carrageenan concentration in the wells (600 μg/mL) was obtained by combining 0.5 mL of Sample 2 with the same volume of culture media. Stock solutions with 120, 12, and 1.2 μg/mL iota-carrageenan concentrations were obtained from this stock solution through 1:10 serial dilutions using Sample P2 as diluent. The corresponding final concentrations of iota-carrageenan in the wells (60, 6, and 0.6 μg/mL) were obtained by applying 0.5 mL of each of these dilutions with the same volume of culture media.

All concentrations and control were tested in triplicate.

#### Preparation of formulations from Samples 3 and P3

The highest iota-carrageenan concentration in the wells (600 μg/mL) was obtained by applying 0.5 mL of Sample 3 with the same volume of culture media. Stock solutions with 120, 12, and 1.2 μg/mL iota-carrageenan concentrations were obtained from this stock solution by 1:10 serial dilutions using Sample P2 as diluent. The corresponding final concentrations of iota-carrageenan in the wells (60, 6, and 0.6 μg/mL) were obtained by applying 0.5 mL of each of these dilutions with the same volume of culture media.

All concentrations and control were tested in triplicate.

To determine antiviral efficacy of formulations through titer reduction assay, sample formulations were used at a final iota-carrageenan concentration of 600 μg/mL, 60 μg/mL, 6 μg/mL, and 0.6 μg/mL. Equivalent amounts of diluents (Samples P1, P2, and P3) were used for titer reduction assay as controls for the corresponding sample formulations.

### Titer reduction assay

Vero E6 cells were seeded in 12-well plates at a density of 2.5x10^5^/well and grown overnight at 37^˚^C under 5%CO_2_.

The next day, cells were washed with Dulbecco’s Phosphate-Buffered Saline (DPBS), pH 7.2, followed by addition of equivalent amount of c-MEM supplemented with reduced fetal bovine serum (2%) and sample formulations/diluents. Formulations and diluents were incubated with cells for two hours, after which the supernatant was removed.

Cells were infected with 2.5x10^4^ pfu (MOI = 0.1) of virus for 1 hour at 37˚C, 5%CO_2_ with rocking at 15-minute intervals. After incubation, wells were washed with DPBS, and sample formulations / diluents were added at the same concentrations.

After incubation for two days, well contents were collected, and titer was determined.

Residual virus was measured in the different supernatants, by TCID50 assay in 96-well plate format with 3 wells per dilution of virus. 10-fold serial dilutions (10^−1^ to 10^−7^) of collected samples (treated, control, or virus only) were used to infect Vero E6 cells in a 96-well plate. The cell plates were incubated at 37°C, 5% CO2, with humidity for an additional 2 days. After 2 days, the virus endpoint titer was determined using the Reed-Muench formula and expressed as log TCID50/mL using MTT proliferation assay to measure cell viability. Virus endpoint titer was determined using the Reed-Muench formula and expressed as log TCID_50_/mL [32].

### Statistical analysis of data

Medians of virus titer obtained after each treatment were calculated. Each set of formulations containing the same diluent were analyzed separately. Virus titers expressed in TCID_50_/mL were determined after each treatment and with no treatment (untreated or ‘virus only’ wells) for each diluent and compared using the Kruskal Wallis non-parametric test. Pairwise post hoc comparisons were assessed with the Conover test using Bonferroni correction.

All statistical tests were two-sided, and the null hypothesis was rejected at a significance level of 5% (a = 0.05). No mathematical transformation of data was applied. Values of TCID_50_/mL below the limit of detection were considered equal to this limit (31.6 TCID_50_/mL) for all statistical tests and descriptive statistics.

In the cellular viability assays, the results were analyzed statistically by One-way ANOVA followed by Tukey’s multiple comparisons test, using GraphPad Prism version 9.1.0, GraphPad Software, San Diego, California USA, www.graphpad.com.

## Results

To examine the antiviral effects of iota-carrageenan on SARS-CoV-2, three sample formulations were developed and tested. Each of the three sample formulations were tested in a dose dependent manner based on varying concentrations of iota-carrageenan ranging from 600 μg/mL to 0 μg/mL.

**Fig 1:**
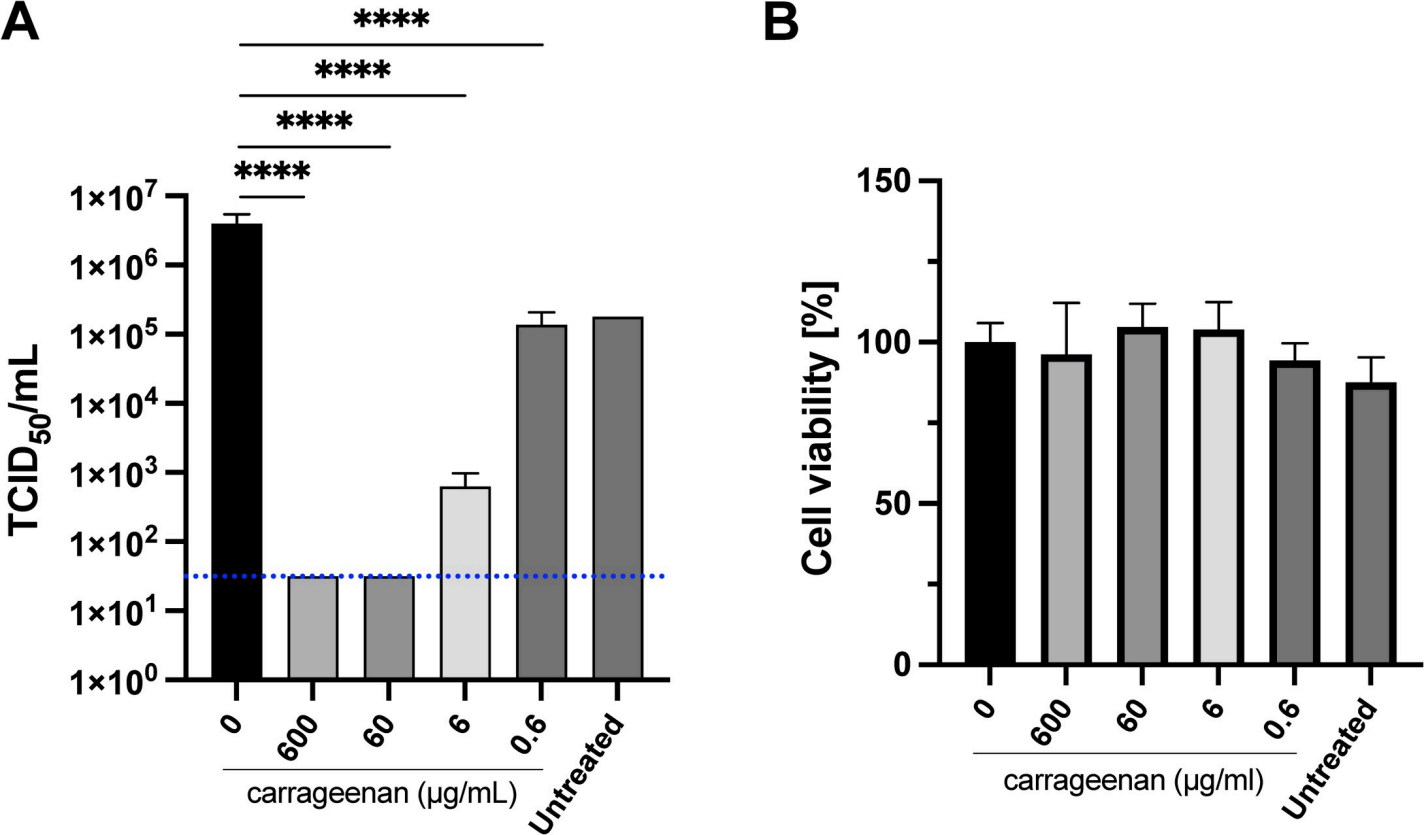
SARS-CoV-2 viral titer after treatment with Samples 1 and P1. A) Infection assay. Sample 1 composition: 1.2mg/mL iota-carrageenan, 9 mg/mL sodium chloride, pH 6–7. Vero E6 were pre-treated for two hours with dilutions of Sample 1 with Sample P1 (diluent without iota-carrageenan) to obtain 600 μg/mL, 60 μg/mL, 6 μg/mL, and 0.6 μg/mL iota-carrageenan final concentration. After this pretreatment, cells were infected with SARS-CoV-2 and incubated for 48 hours in the presence of the same dilutions of Sample 1. Supernatants were harvested and virus yield was determined using an end point dilution assay (TCID_50_). Controls consisted of untreated infected cells or infected cells treated with P1 (no iota-carrageenan). Results were determined using the Reed and Muench formula and expressed as log TCID_50_/mL. The dotted line shows the limit of detection (LOD). Testing of samples was performed in triplicate, and the p-values are p≤0.00025 (****). B) Cellular viability assays. Vero-E6 cells were treated with iota-carrageenan or vehicle (600 μg/mL to 0 μg/mL) for 48 h at 37°C. After incubation, cellular viability was analyzed, and no statistically significant difference was found between the groups compared to the untreated control group (Group 600 μg/ml, p = 0.7464, Group 60 μg/ml, p = 0.0908, Group 6 μg/ml, p = 0.1208, and Group 0.6 μg/ml, p = 0.8938). Data are expressed as mean ± SD. Therefore, these compositions do not adversely affect cell viability. For this reason, cell lysis and death detected in the reported experiments after infection must be attributed to the action of the virus.

The medians of the virus titer found after each treatment with iota-carrageenan solutions in diluent P1 (sodium chloride 9 mg/mL adjusted to pH 6–7) showed that iota-carrageenan markedly inhibits SARS-CoV-2 production in a dose-dependent manner, Fig 1A. In other set of experiments, Vero E6 cells were treated with different iota-carrageenan concentrations (600 μg/mL to 0.6 μg/ml), and cell viability was quantified, No difference in cell viability was observed in iota-carrageenan treated cells compared to vehicle-treated control cells, without the addition of virus, Fig 1B.

SARS-CoV-2 samples treated with 600 μg/mL,60 μg/mL and 6 μg/mL of Sample 1 significantly reduced the residual virus titer when compared to untreated control and Diluent P1 at a concentration not below 6 μg/mL. No statistically significant reductions of the virus were detected after treatment with 0.6 μg/mL of iota-carrageenan. Treatment with Diluent P1 demonstrated a statistically significant increase in residual virus titer when compared with untreated control. This could be due to either better growth conditions for the virus or to an analytical artifact but does not influence the main conclusion, as Diluent P1 does not have any antiviral activity, Fig 1A. Taking the untreated residual virus titer as a reference, SARS-CoV-2 titer is reduced ca. 4 logarithmic units (more than 3.75, in fact) after treatment with 600 or 60 μg/mL iota-carrageenan solutions containing 9 mg/mL sodium chloride adjusted to pH 6–7. In the same diluent SARS-CoV-2 titer is reduced more than 2 logarithmic units after treatment with a solution containing 6 μg/ml iota-carrageenan.

**Fig 2:**
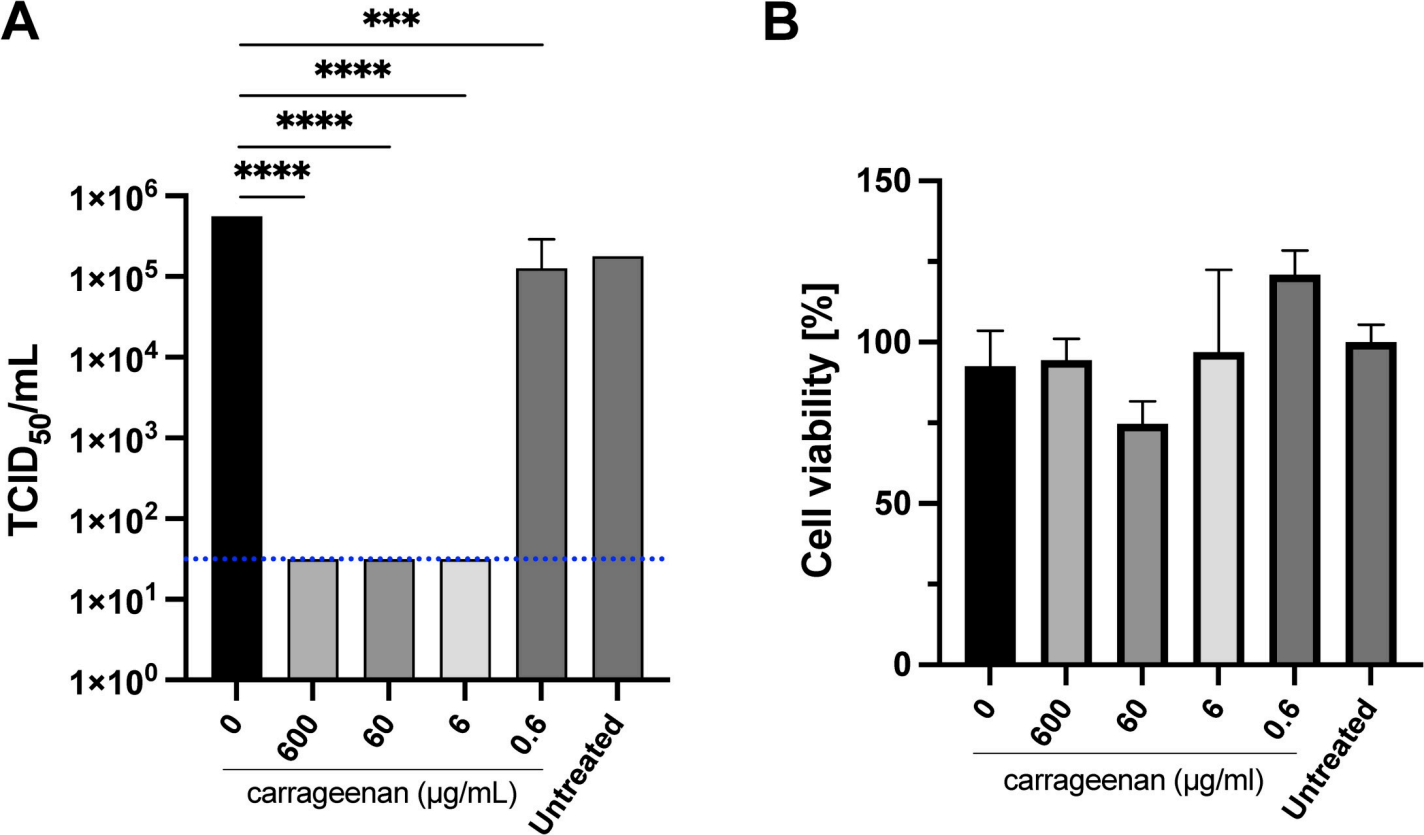
SARS-CoV-2 viral titer after treatment with Samples 2 and P2. A) Infection assay. Sample 2 composition: 1.2mg/mL iota-carrageenan, 5 mg/mL sodium chloride, pH 6–7. Vero E6 were pre-treated with dilutions of Sample 2 with Sample P2 (diluent without iota-carrageenan) to get 600 μg/mL, 60 μg/mL, 6 μg/mL, and 0.6 μg/mL final iota-carrageenan concentration for two hours. After this pretreatment, cells were infected with SARS-CoV-2 and incubated for 48 hours in the presence of the same dilutions of Sample 2. Supernatants were harvested and virus yield was determined using an end point dilution assay (TCID_50_). Controls consisted of untreated infected cells or infected cells treated with P2 (no iota-carrageenan). Results were determined using the Reed and Muench formula and expressed as log TCID_50_/mL. The dotted line shows the limit of detection (LOD). Testing of samples was performed in triplicate, and the p-values are p≤0.00074 (***) and p≤0.00001. B) Cellular viability assays. Vero-E6 cells were treated with iota-carrageenan or vehicle (600 μg/mL to 0 μg/mL) for 48 h at 37°C. After incubation, cellular viability was analyzed, and no statistically significant difference was found between the groups compared to the untreated control group (Group 600 μg/ml, p = 0.9880, Group 60 μg/ml, p = 0.0683, Group 6 μg/ml, p = 0.9993, and Group 0.6 μg/ml, p = 0.1957). Data are expressed as mean ± SD.

The medians of the virus titer found after each treatment with iota-carrageenan solutions when using diluent 2 (sodium chloride 5 mg/mL adjusted to pH 6–7) are shown in Fig 2A. The statistical analysis of data comparing virus titers after each treatment against those of untreated wells (virus only wells) and those treated with Diluent 2 without iota-carrageenan. No difference in cell viability was observed in iota-carrageenan treated cells compared to vehicle-treated control cells, without the addition of virus, Fig 2B.

SARS-CoV-2 samples treated with dilutions of Sample 2 at final iota-carrageenan concentrations of 600 μg/mL, 60 μg/mL, and 6 μg/mL, demonstrated statistically significant reductions in virus titers when compared with untreated controls and controls treated with Diluent P2. However, concentrations of not less than 6 μg/mL showed a much higher logarithmic reduction in virus titer (more than 4 logarithmic units), than 0.6 μg/mL, which showed a reduction below 1 logarithmic unit. As shown in Fig 2A, which plots the logarithm of the residual virus titer in TCID_50_/mL after each treatment. No significant differences were detected between diluent and untreated samples showing that it has no effect on virus titer. This means that the iota-carrageenan is the component that inhibits SARS-CoV-2. In summary, solutions of iota-carrageenan in Diluent P2 (sodium chloride 5 mg/mL and pH adjusted to 6–7) revealed a marked antiviral activity against SARS-CoV-2 in concentrations not below 6 μg/mL and to a lesser extent at lower concentration.

Regarding cell viability, Fig 2B, shows no significant effect on cell viability of diluent P2 and solutions obtained using sample 2 at final iota-carrageenan concentrations of 600 μg/mL and 6 μg/mL. Only two compositions differ significantly from the value obtained on untreated cells. The only case in which cell viability decreased significantly after this treatment is at a concentration of 60 μg/mL of iota-carrageenan. This finding is atypical and does not show any relation to the remaining experimental data. Neither 0.5% sodium chloride (diluent P2) nor iota-carrageenan show any potential to damage Vero cell cultures in any other case. Therefore, this atypical result does not affect the general conclusion about these compositions in that they do not seem to affect cell viability. For this reason, cell lysis and death detected in the reported experiments after infection must be attributed to the action of the virus.

**Fig 3:**
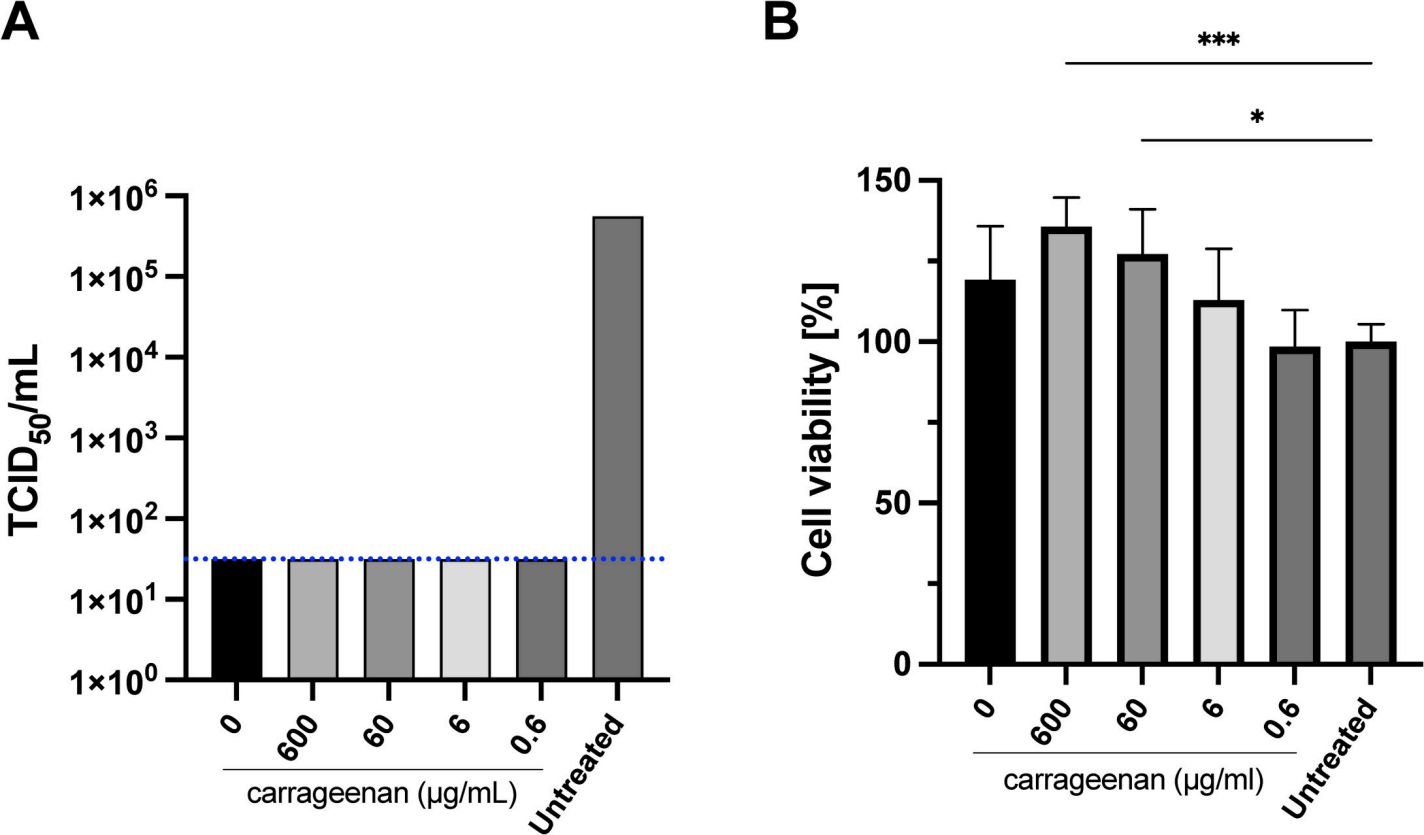
SARS-CoV-2 viral titer after treatment with Samples 3 and P3. A) Infection assay. Sample 3 composition: 1.2mg/mL iota-carrageenan, 5% m/V xylitol, pH 6–7. Vero E6 were pre-treated with dilutions of Sample 3 and Sample P3 (placebo without iota-carrageenan) to get 600 μg/mL, 60 μg/mL, 6 μg/mL, and 0.6 μg/mL final iota-carrageenan concentration for two hours. After this pretreatment, cells were infected with SARS-CoV-2 and incubated for 48 hours in the presence of the same dilutions of Sample 3. Supernatants were harvested, and virus yield was determined by an endpoint dilution assay (TCID_50_). Controls consisted of untreated infected cells or infected cells treated with P3 (no iota-carrageenan). Results were determined using the Reed and Muench formula and expressed as log TCID_50_/mL. The dotted line shows the limit of detection (LOD). Testing of samples was performed in triplicate and the p-value indicates that the groups are significantly different p = 0.0045, nevertheless no post-hoc analysis could be performed. B) Cellular viability assays. Vero-E6 cells were treated with iota-carrageenan or vehicle (600 μg/mL to 0 μg/mL) for 48 h at 37°C. After incubation, cellular viability was analyzed. No statistically significant difference was found between compositions at low iota-carrageenan concentrations, 6 μg/mL (p = 0.6904) and 0.6 μg/mL (p > 0.9999) compared to the untreated control group (Group 6 μg/ml, p = 0.6904, and Group 0.6 μg/ml, p > 0.9999). Compositions with higher concentrations of iota-carrageenan tend to show a significant difference increasing cell viability (Group 600 μg/ml, p = 0.0031 (***), Group 60 μg/ml, p = 0.0417 (*), They are certainly not toxic, but may exert some cytoprotective effect.). Data are expressed as mean ± SD.

The medians of the virus titer found after each treatment with iota-carrageenan solutions in Diluent 3 (xylitol 50 mg/mL adjusted to pH 6–7) are shown in Fig 3A. The statistical analysis of data comparing virus titers after each treatment clearly indicates significant differences between the untreated control and the iota-carrageenan treated groups. No post hoc analysis could be performed.

All final concentrations tested (600–0.6 μg/mL) with iota-carrageenan solutions in Diluent 3 (xylitol 50 mg/mL adjusted to pH 6–7) demonstrated statistically significant antiviral activity including Diluent P3, which did not contain iota-carrageenan. In all these groups the residual viral titer was below the limit of detection. It is important to point out that SARS-Cov-2 residual titer determined in these untreated wells is similar to those obtained in untreated controls in the previous test runs with Diluents P1 and P2. These unexpected results suggest that xylitol has an antiviral activity of its own, as it is the only diluent component that is not present in Diluents P1 and P2. Fig 3 shows a plot of the logarithm of residual virus titers after each treatment.

Regarding cell viability, diluent P3, as well as compositions obtained with sample 3 at low iota-carrageenan concentrations, 6 μg/mL (p = 0.6904) and 0.6 μg/mL (p > 0.9999) do not show any significant effect. However, higher iota-carrageenan concentrations tend to show consistently higher optical densities. These compositions are certainly not toxic to the cell culture but may exert some cytoprotective effect. Therefore, it can be concluded that these compositions do not damage cells, and some of them may have some cytoprotective effect. For this reason, cell lysis and death detected in the reported experiments after infection must be attributed to the action of the virus.

A comparison of all three formulations tested containing 600 μg/mL, 60 μg/mL, and 6 μg/mL iota-carrageenan in solutions with Diluents P1, P2, and P3 show a marked antiviral activity against SARS-CoV-2. In Diluent 2, even a 0.6 μg/mL showed a minor antiviral activity, rendering a residual virus titer significantly less than untreated samples. These data are consistent with previous reported in literature observing that the antiviral action of iota-carrageenan is due to the electrostatic attraction between its negatively charged molecules and positively charged viral particles. The electric attraction force between opposite charges decreases as ionic strength increases. For this reason, iota-carrageenan is speculated to be a more potent antiviral in media with lower ionic strength. Solutions in Diluent 3, which contained xylitol, were the most effective and demonstrated an antiviral effect at all concentrations tested. The only composition which showed statistically significant reduction in cell viability was a solution containing 0.5% w/v sodium chloride and 60 μg/mL of iota carrageenan but without relation to any other experimental data. Even though this solution is hypotonic, other solutions with lower and higher iota carrageenan concentrations did not show any reduction in cell viability. This atypical result could be due to some variation in well location or other experimental factors and does not affect the overall conclusion about iota carrageenan and 0.5% sodium chloride in that they do not adversely affect cell viability. Compositions containing xylitol and high concentrations of iota carrageenan (600 μg/mL and 60 μg/mL) may have some cytoprotective effect.

## Discussion

Results from our study indicate that iota-carrageenan significantly inhibits SARS-CoV-2 *in vitro*. Our results are in line with those already published in the literature [33, 34] and show promise for the clinical use of an iota-carrageenan nasal spray for the prevention and early treatment of COVID-19. Iota-carrageenan nasal spray formulations, already effective *in vitro* against rhinovirus [15], proved to be clinically effective in preventing and reducing the symptoms and duration of the common cold [9–11, 19]. Moreover, the concentrations of iota-carrageenan tested in this study were expected to be similar to the immediate concentration achieved after the administration of a nasal spray. The estimated amount of airway surface liquid volume in the nasal cavity is in the range of 50–375 μL, based on data reported by different sources. On one hand, these reported values of the surface area of the nasal mucosa range from 100 to 250 cm^2^ [35–38]. On the other hand, reported values of the airway surface liquid height are within the range of 5–15 μm [39, 40]. If we take an average of 200 μL of airway surface liquid in the nose (i.e., an average airway surface liquid height of 10 μm and an average nasal mucosa surface area of 200 cm^2^) plus 200 μL of formulation after delivering one 100-μL of a 1.2 mg/mL iota-carrageenan solution in each nostril, the immediate concentration of iota-carrageenan in the nasal cavity would be 600 μg/mL. This coincides with the highest concentration tested *in vitro* and capable of reducing virus titer to the LOD in our assay. Furthermore, considering that even 1/100 of this concentration is still active *in vitro* and that iota-carrageenan may stay for up to four hours [20] in the nasal cavity, we can reasonably surmise that this nasal spray may significantly help in the prevention and early treatment of COVID-19. Expected concentrations of iota-carrageenan in the nasal cavity would be even higher if we consider a nasal formulation containing 1.7 mg / mL (0.17% m/V) as some marketed nasal sprays already have.

As already commented in the introduction, a study in New Zealand rabbits tested the intranasal safety of iota-carrageenan up to daily doses of 448 g/kg. The estimated maximum daily dose of a nasal spray, when applied one actuation in each nostril 6 times a day, would be: 12 x 0.17 mg/actuation = 2.04 mg/day, which is equivalent to 29 μg/kg, and 15 times less than the maximum intranasal dose already tested in New Zealand rabbits for 28 days [26].

Another important finding from our study is that xylitol may exert an antiviral effect on SARS-CoV-2. Xylitol can reduce Human Respiratory Syncytial Virus titers in Hep-2 cell culture and infected mice [25]. Xylitol solutions at 50 mg/mL (ca. 329 mM) are practically isotonic [28] and have proved to be safe for use in the nasal and inhalation administration routes [24, 27, 28], suggesting that using iota-carrageenan and xylitol in combination might be a good strategy for a nasal spray formulation. Further testing would be needed to better characterize the antiviral action of xylitol and its combination with iota-carrageenan.

Despite the implementation of significant personal protection measures, the COVID-19 pandemic continues to affect a significant proportion of health care workers with severe consequences for them, their patients, and the wider community. At the same time, most COVID-19 patients remain at home, thus increasing the likely exposure of other household members and caregivers. Providing these groups with simple interventions, such as nasal sprays with either iota-carrageenan and/or xylitol in nasal devices, may lower the risk of infection progression and transmission.

An inhalation solution of the same composition may be effective in severe cases of COVID-19. While there are studies showing the safety of both carrageenan and xylitol use through nebulization [26, 28], clinical trials would be needed to fully confirm these hypotheses. Risk of spreading the virus should be considered in this form of administration and due protection should be used to contain it [41].

Randomized, double-blind, placebo-controlled clinical trials are needed to confirm that the *in vitro* results obtained in our experiments correlate *in vivo*. A pilot study has been recently published showing a significant reduction in the risk of transmission in healthy hospital personnel dedicated patients when using an iota-carrageenan nasal spray corresponding to the composition of sample 1 of our experiment (1.7 mg/mL iota-carrageenan in 0.9% sodium chloride) in addition to standard preventive measures (ClinicalTrials.gov Identifier: NCT04521322) [42].

## Conclusions

Iota-carrageenan inhibits SARS CoV-2 *in vitro* at concentrations easily achievable by nasal and nebulization formulations. Furthermore, xylitol may also exhibit antiviral activity on SARS-CoV-2, but further testing would be needed to characterize its antiviral action. A combination of iota-carrageenan and xylitol may increase the benefit of a formulated nasal spray. A randomized, double-blind, placebo-controlled clinical trial has recently been published, showing the efficacy of the formulation containing 1.7 mg/mL iota carrageenan in 0.9% sodium chloride in the prevention of transmission of COVID 19 in hospital personnel dedicated to care of COVID-19 patients when taken in addition to standard preventive measures. Further clinical trials are needed to confirm other potential benefits of these formulations in the prevention and early treatment of COVID-19.

## Supporting information

S1 Table Residual virus titer (TCID50/mL) after treatment with iota-carrageenan solutions in Diluent P1 (sodium chloride 9 mg/mL adjusted to pH 6–7). (PDF)

S2 Table Statistical analysis of residual virus titers determined after each treatment with different concentrations of iota-carrageenan in Diluent P1 (sodium chloride 9 mg/mL adjusted to pH 6–7). (PDF)

S3 Table Cell viability found by MTT assay after treatment with diluent P1 and and solutions of iota carrageenan obtained from sample 1 without the addition of virus expressed as optical density and statistical analysis compared to untreated cells. The original data of the residual SARS-CoV-2 viral titer after treatment with iota-carrageenan solutions in Diluent P2 and the viability assay, related to Fig 2A and 2B. respectively.
(PDF)

S4 Table Residual virus titer (TCID_50_/mL) after treatment with iota-carrageenan solutions in Diluent P2 (sodium chloride 5 mg/mL adjusted to pH 6–7). (PDF)

S5 Table Statistical analysis of residual virus titers determined after each treatment with different concentrations of iota-carrageenan in Diluent P2 (sodium chloride 5 mg/mL adjusted to pH 6–7). (PDF)

S6 Table Cell viability found by MTT assay after treatment with diluent P2 and solutions of iota carrageenan obtained from sample 2 without the addition of virus expressed as optical density and statistical analysis compared to untreated cells. The original data of the residual SARS-CoV-2 viral titer after treatment with iota-carrageenan solutions in Diluent P3 and the viability assay, related to Fig 3A and 3B. respectively.
(PDF)

S7 Table Residual virus titer (TCID50/mL) after treatment with iota-carrageenan solutions in Diluent P3 (xylitol 50 mg/mL adjusted to pH 6–7). (PDF)

S8 Table Statistical analysis of residual virus titers determined after each treatment with different concentrations of iota-carrageenan in Diluent P3 (xylitol 50 mg/mL adjusted to pH 6–7). (PDF)

S9 Table Cell viability found by MTT assay after treatment with diluents and solutions of iota carrageenan without the addition of virus expressed as optical density and statistical analysis compared to untreated cells. (PDF)
