# Supplementary material for: Iota-carrageenan and xylitol inhibit SARS-CoV-2 in Vero cell culture
Source: PLoS One. 2021 Nov 19;16(11):e0259943. doi: 10.1371/journal.pone.0259943 (PMC8604354; doi:10.1371/journal.pone.0259943)
Supplement: S1 Table — (PDF) [file pone.0259943.s001.pdf]

Table S1. Residual virus titer ($TCID_{50}$/mL) after treatment with iota-carrageenan solutions in Diluent P1 (sodium chloride 9 mg/mL adjusted to pH 6-7)

Treatment	Residual virus titer ($TCID_{50}$/mL) after treatment with iota-carrageenan solutions in diluent P1 (solidum chloride 9 mg/mL adjusted to pH 6-7)			
	Replicate 1	Replicate 2	Replicate 3	Median
**Untreated**	1.78E+05	1.78E+05	1.78E+05	1.78E+05
**600 µg/mL**	3.16E+01	3.16E+01	3.16E+01	3.16E+01
**60 µg/mL**	3.16E+01	3.16E+01	3.16E+01	3.16E+01
**6 µg/mL**	5.62E+02	3.16E+02	1.00E+03	5.62E+02
**0.6 µg/mL**	1.78E+05	5.62E+04	1.78E+05	1.78E+05
**0 µg/mL (Diluent P1)**	3.16E+06	5.62E+06	3.16E+06	3.16E+06
