# Supplementary material for: Iota-carrageenan and xylitol inhibit SARS-CoV-2 in Vero cell culture
Source: PLoS One. 2021 Nov 19;16(11):e0259943. doi: 10.1371/journal.pone.0259943 (PMC8604354; doi:10.1371/journal.pone.0259943)
Supplement: S2 Table — (PDF) [file pone.0259943.s002.pdf]

**Table S2. Statistical analysis of residual virus titers determined after each treatment with different concentrations of iota-carrageenan in Diluent P1 (sodium chloride 9 mg/mL adjusted to pH 6-7)**

| **Kruskal Wallis test** | Chi-square = 16.646, degrees of freedom = 5, p-value = 0.00522<br>This means that there are statistically significant differences between the treatments ($p < 0.05$) | | | | |
|---|---|---|---|---|---|
| **Treatments used as contrast for Conover test** | **Conover post-hoc test (p-values for contrasts between iota-carrageenan concentrations vs. untreated wells and diluent treated wells without iota-carrageenan)** | | | | |
| | **Diluent (0 µg/mL)** | **0.6 µg/mL** | **6 µg/mL** | **60 µg/mL** | **600 µg/mL** |
| **Untreated** | 0.002 | 1.00 | 0.00025 | 2.8 E-07 | 2.8 E-07 |
| **Diluent P1** | ___ | 0.00025 | 5.1 E-07 | 4.9 E-09 | 4.5 E-09 |
