# Supplementary material for: Iota-carrageenan and xylitol inhibit SARS-CoV-2 in Vero cell culture
Source: PLoS One. 2021 Nov 19;16(11):e0259943. doi: 10.1371/journal.pone.0259943 (PMC8604354; doi:10.1371/journal.pone.0259943)
Supplement: S3 Table — The original data of the residual SARS-CoV-2 viral titer after treatment with iota-carrageenan solutions in Diluent P2 and the viability assay, related to Fig 2A and 2B. respectively. (PDF) [file pone.0259943.s003.pdf]

Table S3. Cell viability found by MTT assay after treatment with diluent P1 and and solutions of iota carrageenan obtained from sample 1 without the addition of virus expressed as optical density and statistical analysis compared to untreated cells

	Diluent 1	600 mg/mL	60 mg/mL	6 mg/mL	0.6 mg/mL	Untreated cells
	0.385	0.382	0.382	0.464	0.365	0.366
	0.392	0.347	0.412	0.41	0.366	0.349
	0.403	0.341	0.421	0.447	0.332	0.308
	0.385	0.364	0.37	0.362	0.336	
	0.391	0.321	0.432	0.409	0.365	
	0.436	0.322	0.462	0.405	0.377	
	0.384	0.527	0.397	0.383	0.392	
	0.347	0.391	0.386	0.373	0.381	
	0.38	0.374	0.406	0.386	0.388	
**Mean**	0.389	0.374	0.408	0.404	0.367	0.341
**p value**	0.02	0.40	0.01	0.02	0.12	
