# Supplementary material for: Iota-carrageenan and xylitol inhibit SARS-CoV-2 in Vero cell culture
Source: PLoS One. 2021 Nov 19;16(11):e0259943. doi: 10.1371/journal.pone.0259943 (PMC8604354; doi:10.1371/journal.pone.0259943)
Supplement: S4 Table — (PDF) [file pone.0259943.s004.pdf]

Table S4. Residual virus titer ($TCID_{50}$/mL) after treatment with iota-carrageenan solutions in Diluent P2 (sodium chloride 5 mg/mL adjusted to pH 6-7)

Treatment	Residual virus titer ($TCID_{50}$/mL) after treatment with iota-carrageenan solutions in diluent P2 (sodium chloride 5 mg/mL adjusted to pH 6-7)			
	Replicate 1	Replicate 2	Replicate 3	Median
Untreated	5.62E+05	5.62E+05	5.62E+05	5.62E+05
600 µg/mL	3.16E+01	3.16E+01	3.16E+01	3.16E+01
60 µg/mL	3.16E+01	3.16E+01	3.16E+01	3.16E+01
6 µg/mL	3.16E+01	3.16E+01	3.16E+01	3.16E+01
0.6 µg/mL	3.16E+05	3.16E+04	3.16E+04	3.16E+04
0 µg/mL (Diluent P2)	5.62E+05	3.16E+05	5.62E+05	5.62E+05
