# Supplementary material for: Iota-carrageenan and xylitol inhibit SARS-CoV-2 in Vero cell culture
Source: PLoS One. 2021 Nov 19;16(11):e0259943. doi: 10.1371/journal.pone.0259943 (PMC8604354; doi:10.1371/journal.pone.0259943)
Supplement: S5 Table — (PDF) [file pone.0259943.s005.pdf]

Table S5. Statistical analysis of residual virus titers determined after each treatment with different concentrations of iota-carrageenan in Diluent P2 (sodium chloride 5 mg/mL adjusted to pH 6-7)

**Kruskal Wallis test**	Chi-square = 16.55, degrees of freedom = 5, p-value = 0.00543<br>This means that there are statistically significant differences between the treatments (p < 0.05)				
**Treatments used as contrast for Conover test**	**Conover post-hoc test (p-values for contrasts between iota-carrageenan concentrations vs. untreated wells and diluent treated wells without iota-carrageenan)**				
	**Diluent (0 µg/mL)**	**0.6 µg/mL**	**6 µg/mL**	**60 µg/mL**	**600 µg/mL**
**Untreated**	1.00	0.00066	1.1 E-07	1.1 E-07	1.1 E-07
**Diluent P2**	___	0.0074	3.9 E-07	3.9 E-07	3.9 E-07
