# Supplementary material for: Iota-carrageenan and xylitol inhibit SARS-CoV-2 in Vero cell culture
Source: PLoS One. 2021 Nov 19;16(11):e0259943. doi: 10.1371/journal.pone.0259943 (PMC8604354; doi:10.1371/journal.pone.0259943)
Supplement: S6 Table — The original data of the residual SARS-CoV-2 viral titer after treatment with iota-carrageenan solutions in Diluent P3 and the viability assay, related to Fig 3A and 3B. respectively. (PDF) [file pone.0259943.s006.pdf]

Table S6. Cell viability found by MTT assay after treatment with diluent P2 and solutions of iota carrageenan obtained from sample 2 without the addition of virus expressed as optical density and statistical analysis compared to untreated cells

	Diluent 2	600 mg/mL	60 mg/mL	6 mg/mL	0.6 mg/mL	Untreated cells
	0.466	0.386	0.355	0.289	0.51	0.438
	0.317	0.412	0.301	0.314	0.498	0.393
	0.343	0.43	0.361	0.298	0.52	0.414
	0.38	0.436	0.319	0.437	0.538	
	0.339	0.383	0.296	0.278	0.461	
	0.41	0.373	0.281	0.481	0.507	
	0.394	0.369	0.29	0.469	0.449	
	0.394	0.378	0.289	0.526	0.492	
	0.413	0.359	0.298	0.527	0.54	
**Mean**	0.384	0.392	0.310	0.402	0.502	0.415
**p-value**	0.29	0.22	2.2 E -4	0.84	1.4 E-3	
