# Supplementary material for: Iota-carrageenan and xylitol inhibit SARS-CoV-2 in Vero cell culture
Source: PLoS One. 2021 Nov 19;16(11):e0259943. doi: 10.1371/journal.pone.0259943 (PMC8604354; doi:10.1371/journal.pone.0259943)
Supplement: S7 Table — (PDF) [file pone.0259943.s007.pdf]

Table S7. Residual virus titer ($TCID_{50}$/mL) after treatment with iota-carrageenan solutions in Diluent P3 (xylitol 50 mg/mL adjusted to pH 6-7)

Treatment	Residual virus ($TCID_{50}$/mL) after treatment with iota-carrageenan solutions in Diluent P3 (xylitol 50 mg/mL adjusted to pH 6-7)			
	**Replicate 1**	**Replicate 2**	**Replicate 3**	**Median**
**Untreated**	5.62E+05	5.62E+05	5.62E+05	5.62E+05
**600 µg/mL**	3.16E+01	3.16E+01	3.16E+01	3.16E+01
**60 µg/mL**	3.16E+01	3.16E+01	3.16E+01	3.16E+01
**6 µg/mL**	3.16E+01	3.16E+01	3.16E+01	3.16E+01
**0.6 µg/mL**	3.16E+01	3.16E+01	3.16E+01	3.16E+01
**0 µg/mL (Diluent P3)**	3.16E+01	3.16E+01	3.16E+01	3.16E+01
