# Supplementary material for: Iota-carrageenan and xylitol inhibit SARS-CoV-2 in Vero cell culture
Source: PLoS One. 2021 Nov 19;16(11):e0259943. doi: 10.1371/journal.pone.0259943 (PMC8604354; doi:10.1371/journal.pone.0259943)
Supplement: S8 Table — (PDF) [file pone.0259943.s008.pdf]

Table S8. Statistical analysis of residual virus titers determined after each treatment with different concentrations of iota-carrageenan in Diluent P3 (xylitol 50 mg/mL adjusted to pH 6-7)

**Kruskal Wallis test**	Chi-square = 17, degrees of freedom = 5, p-value = 0.0045<br>This means that there are statistically significant differences between the treatments ($p < 0.05$)
