# Supplementary material for: Iota-carrageenan and xylitol inhibit SARS-CoV-2 in Vero cell culture
Source: PLoS One. 2021 Nov 19;16(11):e0259943. doi: 10.1371/journal.pone.0259943 (PMC8604354; doi:10.1371/journal.pone.0259943)
Supplement: S9 Table — (PDF) [file pone.0259943.s009.pdf]

Table S9. Cell viability found by MTT assay after treatment with diluents and solutions of iota carrageenan without the addition of virus expressed as optical density and statistical analysis compared to untreated cells.

	Diluent 3	600 mg/mL	60 mg/mL	6 mg/mL	0.6 mg/mL	Untreated cells
	0.545	0.546	0.511	0.405	0.436	0.438
	0.534	0.543	0.513	0.414	0.389	0.393
	0.526	0.546	0.506	0.366	0.396	0.414
	0.566	0.549	0.494	0.536	0.371	
	0.543	0.542	0.54	0.455	0.389	
	0.519	0.608	0.569	0.478	0.376	
	0.376	0.631	0.651	0.501	0.523	
	0.425	0.588	0.526	0.495	0.408	
	0.419	0.515	0.441	0.57	0.389	
**Mean**	0.495	0.563	0.528	0.469	0.409	0.415
**p-value**	0.08	8.3 E-5	0.01	0.20	0.83	
